# Metabolic syndrome as an indicator of high cardiovascular risk in patients with diabetes: Analyses based on Korea National Health and Nutrition Examination Survey (KNHANES) 2008

**DOI:** 10.1186/1758-5996-6-98

**Published:** 2014-09-12

**Authors:** Sang Youl Rhee, So Young Park, Jin Kyung Hwang, Jung Il Son, Sang Ouk Chin, Young Seol Kim, Jeong-taek Woo

**Affiliations:** Department of Endocrinology and Metabolism, Kyung Hee University School of Medicine, 1 Hoegi-dong, Dongdaemoon-gu, Seoul, 130-702 South Korea

**Keywords:** Metabolic syndrome, Syndrome X, Cardiovascular disease, Framingham risk score, Diabetes mellitus, DM, Dyslipidemia, Cholesterol, KNHANES, Korea

## Abstract

**Background:**

Patients with either diabetes mellitus (DM) or metabolic syndrome (MS) are recognized as a high risk group for cardiovascular disease (CVD). Several studies have demonstrated the clinical value of MS for predicting additional CVD risk in the DM population, although the clinical significance remains debatable.

**Methods:**

We used the Korea National Health and Nutrition Examination Survey (KNHANES) 2008, which is the national representative database. We classified the KNHANES subjects based on MS and glucose tolerance status, and compared clinical characteristics and future CVD risk among the subgroups.

**Results:**

A total of 796 of the 4314 subjects were diagnosed with MS. Their clinical characteristics were significantly different from patients without MS. Prevalence of DM was 9.5% in subjects with MS, but only 1.1% in subjects without MS. In addition, there was no MS in 30.9% of total DM patients who were enrolled in this study. For the normal and impaired fasting glucose subgroups, the prevalence of moderate (5–10%) and high (>10%) CVD risk was significantly higher in patients with MS than in patients without MS (*p* < 0.001). However, in the DM subgroup, even after multiple adjustments, there were no differences in clinical characteristics or in the prevalence of moderate to high CVD risk according to MS status. That said, LDL cholesterol in the DM group without MS was significantly higher than in the DM group with MS (*p* = 0.010).

**Conclusions:**

The efficacy of MS as a screening tool for high CVD risk may be limited in DM patients, and conventional risk factors such as LDL may be more important.

**Electronic supplementary material:**

The online version of this article (doi:10.1186/1758-5996-6-98) contains supplementary material, which is available to authorized users.

## Background

The term “metabolic syndrome” (MS) refers to a cluster of metabolic risk factors that accelerate the development of cardiovascular disease (CVD) [1]. Since Reaven described “syndrome X” in 1988 [2], several expert groups have endeavored to identify diagnostic criteria that will more easily identify patients with multiple risk factors for MS [3]. The most widely recognized metabolic risk factors consist of abdominal obesity; atherogenic dyslipidemia such as elevated triglycerides (TG), apolipoprotein B-containing lipoproteins and low levels of high-density lipoprotein (HDL) cholesterol; elevated blood pressure and glucose levels; and prothrombotic and proinflammatory states [1, 4, 5]. The mechanism of interaction among these metabolic risk factors is not completely understood. However, these factors appear to be associated with metabolic susceptibility (e.g. insulin signaling defects, adipose tissue disorder, mitochondrial dysfunction, endocrine dysfunction, ethnic variations, aging, and drugs), and people who have metabolic susceptibility usually manifest insulin resistance [6].

According to a recent meta-analysis, the risk of CVD in MS subjects is approximately double that of non-MS subjects [4, 7]. Therefore, aggressive interventional strategies for CVD prevention should be initiated earlier in MS subjects. However, there is still debate regarding the value of MS as an additive risk factor for CVD in those who have already been diagnosed with diabetes or have a prediabetic conditions, such as impaired fasting glucose (IFG) [8].

Our aim in this study was to investigate the association of MS and future CVD risk based on glucose tolerance status. Furthermore, we attempted to identify practical methods for screening diabetes patients who are at high risk for CVD using the nationally representative Korean population data base, the Korea National Health and Nutrition Examination Survey (KNHANES) of 2008.

## Methods

### KNHANES

KNHANES is a nationwide, population-based, and cross-sectionally designed health survey conducted by the Korea Centers for Disease Control and Prevention. After the first KNHANES was conducted in 1998, the second, third, and fourth surveys were conducted in 2001, 2005, and 2007–2009, respectively.

We utilized the KNHANES 2008 data in this study. KNHANES 2008 was conducted from January to December 2008. The subject population included all households recorded in the 2005 Population and Housing Census in Korea. Relevant households were randomly selected through stratified and multistage probability sampling. As rolling survey methods were used for sampling, the sample for each year was a probability sample representing all parts of the country.

### Study subjects

Subjects aged 20 years and older were selected from the KNHANES 2008 sample (*n* = 6,123). Of these 6,123 subjects, individuals taking medication for hypertension, Diabetes Mellitus, or dyslipidemia (*n* = 1,303) were excluded to eliminate the potential effect of medication on MS status, as were those with insufficient data to diagnose MS (*n* =197). Subjects who had a chronic disease including any type of cancer, viral hepatitis carrier, liver cirrhosis, current tuberculosis, chronic obstructive lung disease, renal failure, myocardial infarction, angina or stroke were also excluded (*n* = 168). Subjects lacking sufficient demographic data, including age, sex, body mass index (BMI), physical activity, smoking and drinking history, income level, education level, residential district and occupation were also excluded (*n* = 141). All subjects included in the study had blood sample data. A total of 4,314 subjects were enrolled in this study.

### Study Methods

The subjects were divided according to presence of MS and glucose tolerance status. The Modified National Cholesterol Education Program Adult Treatment Panel III (NCEP-ATP III) criteria were adopted and MS was diagnosed if a subject met three or more of the following five factors: elevated waist circumference, TG ≥150 mg/dL, HDL cholesterol <40 mg/dL in males and <50 mg/dL in females, systolic blood pressure ≥130 mmHg and/or diastolic ≥85 mmHg, and fasting glucose ≥100 mg/dL [9]. However, the criterion of waist circumference measurement was selected based on the Korean Society for the Study of Obesity criteria, as ≥90 cm in males and ≥85 cm in females [10]. The ten-year CVD risk of subjects was also analyzed by age, smoking status, total cholesterol, HDL cholesterol, and systolic blood pressure using the Framingham risk score [11, 12]. Based on these risk criteria, subjects were classified into low (<5%), moderate (5 to <10%) and high-risk (≥10%) groups. Glucose tolerance status was subdivided into normal fasting glucose (NFG), impaired fasting glucose (IFG) and DM based on the fasting plasma glucose concentration and the diagnostic criteria of the American Diabetes Association [13]. Current smokers were defined as those who had smoked more than five packs of cigarettes during their lifetime and were smoking at the time of the survey. All other subjects were defined as non-smokers. Regular alcohol drinkers were those who currently drank alcohol more than one glass per month regardless of alcohol types, and all others were defined as non-drinkers. The sixteen residential areas of the KNHANES were classified into urban or rural areas. Household income was divided into quartiles. Educational status was categorized as none, elementary school, middle school, high school, or college or higher. Occupation was divided into seven groups: group 1, managers, professionals, technicians and associate professionals; group 2, clerical support workers; group 3, service and sales workers; group 4, skilled agricultural, forestry and fishery workers; group 5, craft and related trades workers, plant and machine operators, and assemblers; group 6, elementary occupations; group 7, housewife, student, and unemployed, based on the 6th Korean Standard Classification of Occupations from the Korean National Statistical Office, which was created by following the International Standard Classification of Occupations of the International Labor Organization [14]. Physical activity of the subjects was categorized according to their participation in recreational physical activity during the week prior to the survey.

Samples from all subjects were collected after a >8 hour period of fasting. Specimens were immediately transported to the central laboratory (NeoDIN Medical Institute, Seoul, Korea) where they were analyzed within 24 hours. Biochemical measurements, including total cholesterol, TG, HDL cholesterol, blood urea nitrogen (BUN), creatinine, aspartate aminotransferase (AST), alanine aminotransferase (ALT) and fasting plasma glucose concentration were analyzed using an automated analyzer (Hitachi Automatic Analyzer 7600; Tokyo, Japan) with enzymatic assays. HDL cholesterol was evaluated using standard samples as equivalents between the KNHANES central laboratory and the U.S. Centers for Disease Control and Prevention to produce an accurate lipid profile. The differences between the two laboratories were adjusted for by Passing–Bablok regression [15]. LDL cholesterol levels were calculated using the Friedewald equation [16]. Instead of apolipoprotein, which is typically difficult to analyze in a clinical setting, non-HDL cholesterol was analyzed as a potential predictor of CVD risk in the presence of high serum TG common in subjects with DM [17, 18]. Non-HDL cholesterol was calculated as total cholesterol concentration minus HDL cholesterol. The non-HDL/HDL ratio was divided into tertiles to compare trends of prevalence of IFG and DM in both MS and non-MS subjects. Serum insulin concentration was measured using a gamma counter (1470 Wizard, Perkin Elmer, Turku, Finland) and an immunoradiometric assay (Biosource, Nivelles, Belgium). An updated homeostasis model assessment (HOMA2) method was employed to assess β-cell insulin secretion capacity (HOMA2%B) and insulin sensitivity (HOMA2%S) in fasting plasma glucose and fasting serum insulin [19, 20]. The HOMA2 method, a computerized improvement on the standard HOMA method, reflects a more accurate insulin secretion capacity and insulin resistance than the previous HOMA method [19, 20].

### Statistical analysis

All data are expressed as either numbers with proportions for categorical variables or as means ± SD for numerical variables. Student’s t-tests were used for the comparison of continuous variables and Pearson's Chi-square tests(*χ*^2^-test) were used for the comparison of categorical variables of demographic and clinical characteristics between MS subjects and non-MS subjects. The study population was subdivided into NFG, IFG and DM groups and the prevalence of MS based on glucose tolerance status was analyzed by *χ*^2^-test. In those subgroups, demographic and clinical characteristics and CVD risk were analyzed according to the presence of MS by independent sample *t*-test and *χ*^2^-test. Odds ratios (ORs) and 95% confidence intervals (CIs) for the prevalence of MS according to CVD risk were estimated by logistic regression.

Statistical analyses were conducted using Predictive Analytics Software (PASW; version 18.0) (SPSS, Inc., Chicago, IL, USA). *P*-values less than 0.05 were considered statistically significant.

### Ethics statement

Because this study analyzed publicly available data sets, it was exempt from Institutional Review Board approval.

## Results

### Clinical characteristics

A total of 4,314 subjects were included for analysis in this study. Subjects with MS and those who were classified as non-MS accounted for 18.5% (*n* = 796) and 81.5% (*n* = 3,518), respectively, of the total study population (Additional file 1: Table S1). Mean age, BMI, percentage of males and current smokers were significantly higher in the MS group than in the non-MS group (*p* < 0.001). When compared with MS subjects, non-MS subjects tended to live in urban areas (*p* = 0.041), had a higher household income (*p* < 0.001), and were more educated (*p* < 0.001). Occupations were also significantly different between the study groups (*p* = 0.002). Biochemical parameters, including BUN (*p* < 0.001), creatinine (*p* < 0.001), AST (*p* < 0.001), and ALT (*p* < 0.001) were all significantly higher in MS subjects than in non-MS subjects.

### The prevalence of glucose tolerance status and MS

**Figure 1:**
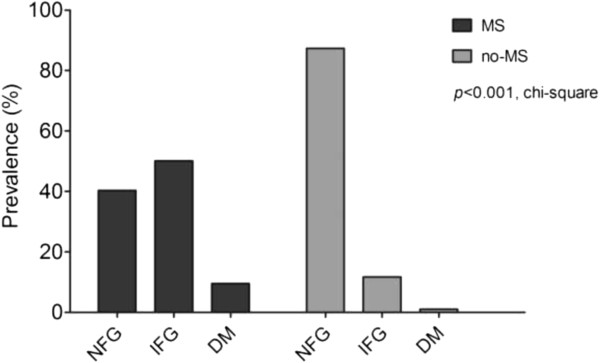
**Prevalence of glucose tolerance status by MS status.**

The prevalence of NFG, IFG and DM in the study sample were 78.6% (n = 3,392), 18.8% (n = 812), and 2.5% (n = 110) respectively. However, there were significant differences in the prevalence of each glucose tolerance status according to MS status (Figure 1). Prevalence of NFG, IFG, and DM in MS subjects was 40.3%, 50.1%, and 9.5% respectively, whereas the prevalence was 87.3%, 11.7%, and 1.0%, respectively, in non-MS subjects (*p* < 0.001). There was a significantly lower prevalence of DM in non-MS subjects, although non-MS subjects comprised 30.9% of the DM subgroup.

### Clinical characteristics according to glucose tolerance status and MS

When comparing subjects by their glucose tolerance status, there were significant differences in clinical variables between MS subjects and non-MS subjects in the NFG and IFG subgroups (Table 1). However, there were no significant differences except for the NCEP-ATP III diagnostic criteria of MS between the MS subjects and non-MS subjects in the DM subgroup. In fact, in the DM subgroup, LDL cholesterol, which is an established risk factor for cardiovascular disease, was significantly higher in non-MS subjects than in MS subjects.

**Table 1:** **Demographic and clinical characteristics of the subjects by glucose tolerance and MS status**

	NFG			IFG			DM		
	MS (-) n = 3,071	MS (+) n = 321	*p*	MS (-) n = 413	MS (+) n = 399	*p*	MS (-) n = 34	MS (+) n = 76	*p*
Age (yrs)	42.1 ± 14.6	50.5 ± 15.5	<0.001	49.4 ± 14.4	50.9 ± 13.0	0.129	51.6 ± 12.7	50.0 ± 13.2	0.536
Sex (M/F, n)	1188/1883	159/162	<0.001	213/200	226/173	0.147	23/11	42/34	0.222
BMI (kg/m^2^)	22.6 ± 2.9	26.3 ± 3.1	<0.001	23.1 ± 2.9	25.9 ± 3.2	<0.001	23.5 ± 3.3	26.1 ± 3.7	0.001
Smoking (n,%)									
No smoker	2353 (76.6)	241 (75.1)	0.535	316 (76.5)	270 (67.7)	0.005	24 (70.6)	50 (65.8)	0.620
Current smoker	718 (23.4)	80 (24.9)		97 (23.5)	129 (32.3)		10 (29.4)	26 (34.2)	
Alcohol (n,%)									
No drinker	1,292 (42.1)	158 (49.2)	0.014	161 (39.0)	153 (38.3)	0.852	13 (38.2)	29 (38.2)	0.994
Regular drinker	1,779 (57.9)	163 (50.8)		252 (61.0)	246 (61.7)		21 (61.8)	47(61.8)	
Location^a^ (n,%)									
Urban area	2,007 (65.4)	189 (58.9)	0.021	263 (63.7)	254 (63.7)	0.995	22 (64.7)	45 (59.2)	0.585
Rural area	1,064 (34.6)	132 (41.1)		150 (36.3)	145 (36.3)		12 (35.3)	31 (40.8)	
Household income^b^ (n,%)									
1st quartile (lowest)	425 (13.8)	78 (24.3)	<0.001	69 (16.7)	64 (16.0)	0.672	9 (26.5)	18 (23.7)	0.522
2nd quartile	773 (25.2)	77 (24.0)		117 (28.3)	100 (25.1)		7 (20.6)	21 (27.6)	
3rd quartile	900 (29.3)	82 (25.5)		117 (28.3)	125 (31.3)		6 (17.6)	19 (25.0)	
4th quartile (highest)	973 (31.7)	84 (26.2)		110 (26.6)	110 (27.6)		12 (35.3)	18 (23.7)	
Education (n,%)									
Elementary school or lower	499 (16.2)	105 (32.7)	<0.001	102 (24.7)	127 (31.8)	0.109	13 (38.2)	25 (32.9)	0.686
Middle school	281 (9.2)	38 (11.8)		50 (12.1)	52 (13.0)		1 (2.9)	6 (7.9)	
High school	1,216 (39.6)	102 (31.8)		163 (39.5)	136 (34.1)		9 (26.5)	24 (31.6)	
College or higher	1,075 (35.0)	76 (23.7)		98 (23.7)	84 (21.1)		11 (32.4)	21 (27.6)	
Occupation^c^ (n,%)									
Group 1	429 (14.0)	40 (12.5)	0.214	42 (10.2)	44 (11.0)	0.783	3 (8.8)	11 (14.5)	0.577
Group 2	269 (8.8)	25 (7.8)		29 (7.0)	29 (7.3)		2 (5.9)	5 (6.6)	
Group 3	398 (13.0)	57 (17.8)		52 (12.6)	52 (13.0)		6 (17.6)	14 (18.4)	
Group 4	266 (8.7)	21 (6.5)		43 (10.4)	50 (12.5)		6 (17.6)	9 (11.8)	
Group 5	295 (9.6)	36 (11.2)		62 (15.0)	68 (17.0)		8 (23.5)	11 (14.5)	
Group 6	259 (8.4)	27 (8.4)		53 (12.8)	42 (10.5)		4 (11.8)	5 (6.6)	
Group 7	1,155 (37.6)	115 (35.8)		132 (32.0)	114 (28.6)		5 (14.7)	21 (27.6)	
Physical activity^d^ (n,%)									
None	1,270(41.4)	153 (47.7)	0.148	172 (41.6)	166 (41.6)	0.098	12 (35.3)	31 (40.8)	0.771
Mild	959 (31.2)	92 (28.7)		117 (28.3)	105 (26.3)		11 (32.4)	19 (25.0)	
Moderate	297 (9.7)	30 (9.3)		37 (9.0)	50 (12.5)		6 (17.6)	11 (14.5)	
Vigorous	545 (17.7)	46 (14.3)		87 (21.1)	78 (19.5)		5 (14.7)	15 (19.7)	
Total cholesterol (mg/dL)	180.9 ± 32.8	202.8 ± 33.9	<0.001	193.7 ± 33.6	201.2 ± 35.8	0.002	206.8 ± 43.1	204.1 ± 35.2	0.732
Triglycerides (mg/dL)	103.9 ± 77.6	251.3 ± 192.9	<0.001	105.7 ± 62.0	217.8 ± 156.2	<0.001	114.1 ± 64.3	256.5 ± 177.5	<0.001
HDL cholesterol (mg/dL)	50.2 ± 10.6	38.9 ± 5.8	<0.001	51.8 ± 10.0	41.4 ± 8.6	<0.001	51.8 ± 10.0	40.8 ± 7.6	<0.001
LDL cholesterol (mg/dL)	110.0 ± 29.9	113.6 ± 44.2	0.145	120.7 ± 31.3	116.2 ± 40.3	0.080	132.2 ± 34.4	112.1 ± 38.5	0.010
Non-HDL cholesterol (mg/dL)	130.7 ± 32.6	163.9 ± 33.0	<0.001	141.8 ± 32.6	159.8 ± 34.4	<0.001	155.0 ± 40.7	163.4 ± 34.4	0.269
Non-HDL/HDL ratio	2.7 ± 1.0	4.3 ± 1.0	<0.001	2.8 ± 0.8	4.0 ± 1.1	<0.001	3.1 ± 0.9	4.1 ± 1.1	<0.001
BUN (mg/dL)	13.7 ± 3.9	14.4 ± 3.9	0.003	15.2 ± 4.6	14.8 ± 3.8	0.143	16.3 ± 4.2	14.9 ± 4.2	0.092
Creatinine (mg/dL)	0.89 ± 0.18	0.95 ± 0.21	<0.001	0.92 ± 0.19	0.93 ± 0.20	0.394	0.89 ± 0.18	0.93 ± 0.24	0.440
AST (IU/L)	20.2 ± 13.1	23.4 ± 11.4	<0.001	22.8 ± 25.2	24.4 ± 11.2	0.234	26.2 ± 21.4	31.7 ± 34.3	0.394
ALT (IU/L)	18.6 ± 14.8	27.4 ± 21.4	<0.001	22.5 ± 43.3	28.7 ± 18.9	0.008	25.9 ± 16.9	34.1 ± 26.7	0.055
Fasting plasma glucose (mg/dL)	88.7 ± 6.0	91.6 ± 5.1	<0.001	105.9 ± 5.9	107.3 ± 6.5	0.001	160.8 ± 43.4	167.0 ± 45.2	0.505
Fasting serum insulin	8.50 ± 3.28	11.53 ± 7.42	<0.001	9.52 ± 5.13	11.99 ± 7.24	<0.001	11.80 ± 9.01	12.92 ± 8.52	0.533
HOMA2%B (%)	104.4 ± 28.7	118.6 ± 40.5	<0.001	78.2 ± 25.5	88.7 ± 31.7	<0.001	45.0 ± 26.9	47.8 ± 31.0	0.641
HOMA2%S (%)	102.7 ± 35.2	79.2 ± 28.7	<0.001	91.9 ± 34.2	77.0 ± 32.7	<0.001	84.2 ± 43.6	68.9 ± 34.0	0.076

by independent *t*-test or chi-square test, mean ± S.D. or n (%).

^a^The 16 residential areas of the KNHANES were classified into two groups: urban areas, including metropolitan cities such as Seoul, Busan, Daegu, Incheon, Gwangju, Daejeon, and Ulsan, as well as metropolitan areas such as Gyeonggi province; rural areas, comprising Gangwon, Chungbuk, Chungnam, Jeonnam, Jeonbuk, Gyeongbuk, Gyeongnam, and Jeju provinces. ^b^Household income was assigned to a category according to the following quartiles: 1st quartile (<bottom 25%), 2nd quartile (25-49%), 3rd quartile (50-75%) and 4th quartile (>top 25%). ^c^Occupation group referred to the KSCO-6 classification. Group 1 indicates managers, professionals, technicians and associate professionals; group 2, clerical support workers; group 3, service and sales workers; group 4, skilled agricultural, forestry and fishery workers; group 5, craft and related trades workers, plant and machine operators, and assemblers; group 6, elementary occupations; group 7, housewife, student, and unemployed. ^d^Physical activity of the subjects was categorized according to their participation in recreational physical activity during the week prior to the survey: none, no or minimal activity; mild, >30 minutes of walking more than 5 days per week; moderate, >30 minutes of physical activity in which the subject was tired or breathing slightly hard compared to normal more than 5 days per week; vigorous, >20 min of vigorous physical activity in which the subject was exhausted or breathing hard compared to normal more than 3 days per week. MS indicates Metabolic syndrome; NFG, normal fasting glucose; IFG, impaired fasting glucose; DM, diabetes mellitus; BMI, body mass index; HDL, high density lipoprotein; LDL, low density lipoprotein; BUN, blood urea nitrogen; AST, aspartate aminotransferase; ALT, alanine aminotransferase; FPG, fasting plasma glucose; HOMA2%B, updated homeostasis model assessment for β-cell insulin secretion; HOMA2%S, updated homeostasis model assessment for insulin sensitivity.

### Risk of a cardiovascular event according to glucose tolerance status and MS

**Figure 2:**
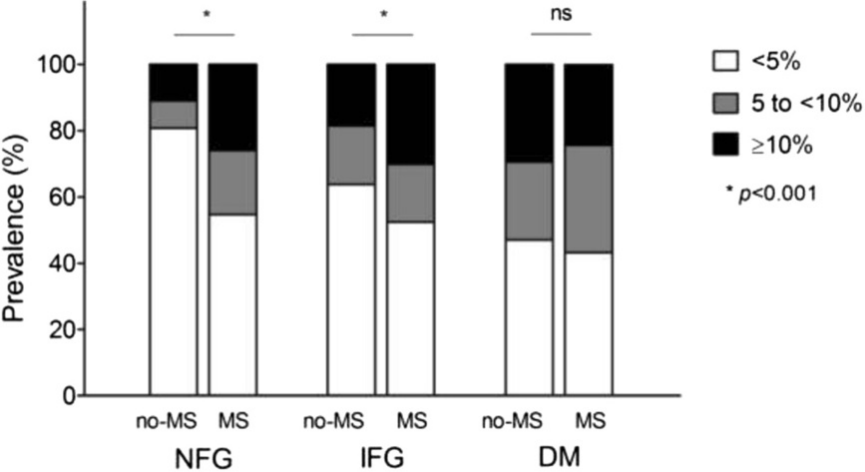
**Ten-year cardiovascular disease risk by glucose tolerance and MS status estimated by Framingham risk score.**

Ten-year CVD event risk was estimated by the Framingham risk score and compared according to glucose tolerance and MS status (Figure 2). Subjects diagnosed with MS had a higher prevalence of moderate (5 to <10%) and high (≥10%) CVD risk than non-MS subjects in the NFG and IFG subgroups (*p* < 0.001). However, in the DM subgroup, the presence of MS was not associated with the prevalence of moderate or high CVD risk (*p =* 0.649). We also analyzed odds ratios for MS prevalence in moderate or high CVD risk subjects by glucose tolerance status, and found that ORs for MS prevalence in moderate or high CVD risk subjects in the DM subgroup were not significant even after multiple adjustments (Table 2).

**Table 2:** **Odds ratio (ORs) and 95% confidence interval (CI) for prevalence of MS in moderate of high CVD risk subgroup by glucose tolerance status**

	Prevalence of MS (n,%)	Adjusted ORs (95% CI)
Total subjects		
Low CVD risk	411 (13.1)	Referent
Moderate CVD risk	154 (31.8)	3.009* (2.405-3.765)
High risk	219 (34.5)	3.502* (2.856-4.295)
NGT subjects		
Low CVD risk	172 (6.6)	Referent
Moderate CVD risk	61 (19.5)	3.549* (2.544-4.952)
High risk	82 (19.9)	3.735* (2.750-5.072)
IFG subjects		
Low CVD risk	207 (44.7)	Referent
Moderate CVD risk	69 (49.3)	1.179 (0.802-1.734)
High risk	119 (61.7)	1.980* (1.390-2.821)
DM subjects		
Low CVD risk	32 (66.7)	Referent
Moderate CVD risk	24 (75.0)	2.367 (0.754-7.437)
High risk	74 (68.5)	1.159 (0.389-3.451)

*p < 0.01. Adjusted for location, household income, and occupation.

*MS* indicates metabolic syndrome, *CVD* cardiovascular disease.

## Discussion

Metabolic syndrome and DM are the most well-known CVD risk factors and are used as a measure of CVD risk in many studies. The pathophysiology of MS is not completely understood, but since insulin resistance is known to be a representative pathophysiological factor, many studies have focused on the relationship between DM and MS [21]. The prevalence of DM risk for patients with MS (diagnosed per NCEP criteria) was 2.99 times higher than patient without MS (95% CI 1.96–4.57) (P for heterogeneity < 0.001) [22]. Approximately 60-70% of DM patients have MS [23, 24]. Previous research indicates that DM and MS are independent CVD risk factors but it is still unclear whether MS is useful as a predictor of CVD risk in subjects with DM [24, 25].

Our study classified the subjects into NFG, IFG and DM subgroups according to glucose tolerance status and analyzed the prevalence of MS and its relationship to CVD risk using the Framingham risk score. CVD risk in MS subjects was higher than in non-MS subjects in the NFG and IFG subgroups, but there was no significant difference in CVD risk between MS subjects and non-MS subjects in the DM subgroup. This suggests that MS is a useful CVD risk predictive factor in the NFG and IFG subgroups, but not in the DM subgroup. On the other hand, LDL cholesterol levels in non-MS subjects in the DM subgroup were higher compared with MS subjects in the DM subgroup. This is likely because DM, a strong CVD risk factor, already existed in the DM subgroup, and the impact of DM was stronger than other components included in the MS cluster.

The term metabolic syndrome refers to a cluster of metabolic risk factors that accelerate the development of cardiovascular disease, and there has been controversy surrounding the establishment of MS diagnostic criteria [3]. Some experts explain that the reason why DM is not included in the NCEP-ATP III criteria (which include BMI, waist circumference, high BP, high TG, low HDL, and high fasting glucose) is because CVD risk associated with MS preferentially increases in subjects with DM [26]. This supports the conclusion of our study that DM has a greater impact on CVD risk than other MS components.

The American Heart Association designates Type 2 DM, together with Peripheral Arterial Disease (PAD) and Carotid Artery Disease (CAD), as “Coronary Heart Disease (CHD) risk equivalents” [5]. In DM patients, hyperglycemia works synergistically with other CVD risk factors such as hypertension, dyslipidemia, obesity, reduced physical activity, and cigarette smoking to increase CVD morbidity and mortality. Due to insulin resistance, plasminogen activator inhibitors and fibrinogen increase and the coagulation process is reinforced so that fibrinolysis is hindered. In addition, DM is related to endothelial, vascular, smooth muscle, and platelet dysfunction [27]. A recent study measured circulating endothelial progenitor cells (EPCs) in CHD and CHD risk equivalent (PAD, CAD and Type 2 diabetes mellitus) groups [28]. EPCs play an important role in neovasculogenesis, vascular repair and atherogenic processes. There were more EPCs in subjects with DM than in other disease groups. These results are another evidence that DM has a strong influence on CVD prevalence. Therefore, independent CVD risk factors such as LDL cholesterol, age, gender, cigarette smoking and family history or parameters such as hs-CRP, CD40L, MCP-1, ICAM-1, VCAM-1, and p-selectin might be helpful in determining CVD risks in individuals with DM [29]. New diagnostic tools should be developed to help correctly predict CVD.

Recently Li et al. studied the relationship between the prevalence of MS and non-embolic ischemic stroke according to glucose tolerance status [30]. The results showed that MS prevalence was higher in the stroke group than in the control group. Subjects were then classified according to glucose tolerance status (NGT, IFG or DM) and a multiple logistic regression analysis was performed. This analysis showed that the ORs of ischemic stroke in the MS with DM, MS with IFG and MS with NGT groups were all high (5.70, 2.24 and 2.19, respectively) (*p* < 0.05). Among them, the odds ratio was the highest in the MS with DM group. Accordingly, they concluded that MS increased ischemic stroke risk, especially in hyperglycemic patients with DM. This contradicts the findings of our study, but this may be due to the fact that they measured blood glucose when a stroke was occurring, and included only acute cerebral stroke patients who visited a university medical center emergency room within 2 hours of the onset of symptoms [30].

Kim et al. studied the association between inflammatory markers, adipokines (hs-CRP, IL-6, resistin, and adiponectin) and MS score. In addition, they explored whether or not MS score is useful in predicting the risk of coronary artery disease in patients with chest pain who received a coronary angiography, according to DM status [31]. Their study findings were similar to ours. MS score was useful in predicting CAD risk in non-DM patients, and an increased MS score was associated with increased IL-6 and decreased adiponectin. However, MS score was not useful for predicting CAD risk in DM patients, nor there was any consistent relationship between MS scores and bioparameters in DM patients. Their study did not completely exclude medication effects such as the effect of anti-diabetic agents, and their work was conducted on patients who likely had CAD and were treated with coronary angiography. On the other hand, our study avoided selection bias and obtained more objective results because it excluded medication effects, used the Framingham risk score as a CVD risk prediction method, and used KNHANES 2008 data to create a large-scale subject group.

This study has several limitations. The first issue is that it is unclear if the Framingham risk score will be an appropriate risk prediction factor for CVD prevalence in the future. In fact, some researchers believe that the Framingham risk score underestimates CVD risk in specific age and gender groups [32, 33]. In addition, more precise methods are available to measure CVD risk, such as the coronary artery calcium score by computed tomography and carotid intima-media thickness by ultrasonography. However, the Framingham risk score is still recognized as a reliable predictor of CVD risk and continues to be used frequently in clinical settings [34]. Carotid intima-media thickness by ultrasonography or coronary artery calcium score by computed tomography (CT) for the purpose of screening is costly, and also may result in unnecessary exposure to contrast media. Another limitation of this study is the cross-sectional design. Therefore, the results of this study cannot provide long-term information about the relationship between MS and CVD risk. Thus, future studies should be long-term and retrospective or prospective in nature. Finally, this study is not widely applicable because it was conducted on Korean subjects only. There are many genetic and lifestyle differences between ethnicities that can greatly impact MS components. Therefore, future studies of replication should include individuals of various ethnic backgrounds.

However, the results of this study are more objective than similarly scaled studies due to the use of national representative epidemiological data, KNHANES, which is collected through strict quality control. Using the KNHANES 2008 database made it possible to gather socioeconomic variables and control and adjust for the effect of these variables on study results. Hence, the results of this study are relatively accurate and detailed analyses were possible.

In addition, our study segmented groups according to glucose tolerance status. This is important because it was possible to independently classify prediabetes patients into the NGT, IGT and DM group according to glucose tolerance status rather than simply creating a DM and a non-DM group. Also, considering that high fasting glucose is an MS component, it is impossible to completely exclude the influence of hyperglycemia on CVD risk in the MS group. However, we could control the influence of hyperglycemia by dividing the MS group according to glucose tolerance status. And we focused on other MS components except high fasting glucose.

In conclusion, MS is not a useful tool for screening CVD risk in DM patients, but may be applicable in NFG or IFG patients. Currently, stress tests such as exercise or adenosine stress SPECT radionuclide myocardial perfusion imaging (rMPI), dobutamine-atropine stress echocardiography and coronary angiography, noninvasive CT coronary angiogram or cardiac magnetic resonance imaging (MRI) techniques have used for screening of cardiovascular disease in DM patients. However, these tests are too invasive, expensive and not accessible. Also, patients could be exposed to radiation and contrast media. Instead, conventional risk factors such as age, gender, cigarette smoking, family history, LDL cholesterol, and inflammation markers may be more important when predicting CVD risk in DM patients. Therefore, MS can be a good tool for non-DM patients, while it is necessary to further study and develop new tools and criteria for risk prediction in DM patients.

## Electronic supplementary material

Additional file 1: Table S1: Demographic and clinical characteristics of the subjects by MS status. (DOCX 21 KB)

## Abbreviations

MS: Metabolic syndrome
CVD: Cardiovascular disease
KNHANES: Korea National Health and Nutrition Examination Survey
NFG: Normal fasting glucose
IFG: Impaired glucose tolerance
DM: Diabetes mellitus
HDL: High density lipoprotein
LDL: Low density lipoprotein
HOMA2%B: Updated homeostasis model assessment for β-cell insulin secretion
HOMA2%S: Updated homeostasis model assessment for insulin sensitivity.
