# Supplementary material for: Metabolic syndrome as an indicator of high cardiovascular risk in patients with diabetes: Analyses based on Korea National Health and Nutrition Examination Survey (KNHANES) 2008
Source: Diabetol Metab Syndr. 2014 Sep 12;6:98. doi: 10.1186/1758-5996-6-98 (PMC4176586; doi:10.1186/1758-5996-6-98)
Supplement: Supplementary file 1 — Additional file 1: Table S1: Demographic and clinical characteristics of the subjects by MS status. (DOCX 21 KB) [file 13098_2014_361_MOESM1_ESM.docx]

**Supplement Table**

|  | MS (-)  n=3,518 | MS (+)  n=796 | *p* |
| --- | --- | --- | --- |
| Age (yrs) | 43.0±14.8 | 50.7±14.1 | <0.001 |
| Sex (Male n, %) | 1,424 (40.5) | 427 (53.6) | <0.001 |
| BMI (kg/m^2^) | 22.6±2.9 | 26.1±3.2 | <0.001 |
| Smoking (n, %) |  |  |  |
| no smoker | 2,693 (76.5) | 561 (70.5) | <0.001 |
| current smoker | 825 (23.5) | 235 (29.5) |  |
| Alcohol (n, %) |  |  |  |
| no drinker | 1,466 (41.7) | 340 (42.7) | 0.590 |
| regular drinker | 2,052 (58.3) | 456 (57.3) |  |
| Location^a^ (n, %) |  |  |  |
| urban area | 2,292 (65.2) | 488 (61.3) | 0.041 |
| rural area | 1,226 (34.8) | 308 (38.7) |  |
| Household income^b^ (n, %) |  |  |  |
| 1^st^ quartile (lowest) | 503 (14.3) | 160 (20.1) | <0.001 |
| 2^nd^ quartile | 897 (25.5) | 198 (24.9) |  |
| 3^rd^ quartile | 1,023 (29.1) | 226 (28.4) |  |
| 4^th^ quartile (highest) | 1,095 (31.1) | 212 (26.6) |  |
| Education (n, %) |  |  |  |
| elementary school or lower | 614 (17.5) | 257 (32.3) | <0.001 |
| middle school | 332 (9.4) | 96 (12.1) |  |
| high school | 1,388 (39.5) | 262 (32.9) |  |
| college or higher | 1,184 (33.7) | 181 (22.7) |  |
| Occupation^c^ (n, %) |  |  |  |
| group 1 | 474 (13.5) | 95 (11.9) | 0.002 |
| group 2 | 300 (8.5) | 59 (7.4) |  |
| group 3 | 456 (13.0) | 123 (15.5) |  |
| group 4 | 315 (9.0) | 80 (10.1) |  |
| group 5 | 365 (10.4) | 115 (14.4) |  |
| group 6 | 316 (9.0) | 74 (9.3) |  |
| group 7 | 1,292 (36.7) | 250 (31.4) |  |
| Physical activity^d^ (n, %) |  |  |  |
| none | 1,454 (41.3) | 350 (44.0) | 0.098 |
| mild | 1,087 (30.9) | 216 (27.1) |  |
| moderate | 340 (9.7) | 91 (11.4) |  |
| vigorous | 637 (18.1) | 139 (17.5) |  |
| Glucose tolerance status |  |  |  |
| normal fasting glucose | 3070 (87.3) | 321 (40.3) | <0.001 |
| impaired fasting glucose | 413 (11.7) | 399 (50.1) |  |
| diabetes mellitus | 34 (1.0) | 76 (9.5) |  |
| Total cholesterol (mg/dL) | 182.7±33.3 | 202.1±35.0 | <0.001 |
| Triglycerides (mg/dL) | 104.2±75.8 | 235.0±174.5 | <0.001 |
| HDL cholesterol (mg/dL) | 50.4±10.6 | 40.4±7.6 | <0.001 |
| LDL cholesterol (mg/dL) | 111.4±30.4 | 114.8±41.7 | 0.032 |
| Non-HDL cholesterol (mg/dL) | 132.3±32.9 | 161.8±33.9 | <0.001 |
| Non-HDL/HDL ratio | 2.8±1.0 | 4.1±1.1 | <0.001 |
| BUN (mg/dL) | 13.9±4.0 | 14.6±3.9 | <0.001 |
| creatinine (mg/dL) | 0.89±0.18 | 0.94±0.21 | <0.001 |
| AST (IU/L) | 20.5±15.2 | 24.7±15.2 | <0.001 |
| ALT (IU/L) | 19.1±20.4 | 28.7±20.8 | <0.001 |
| Fasting plasma glucose (mg/dL) | 91.5±11.4 | 106.7±25.8 | <0.001 |
| Fasting serum insulin | 8.65±3.66 | 11.89±7.45 | <0.001 |
| HOMA2 %B (%) | 100.7±30.0 | 96.8±41.4 | 0.012 |
| HOMA2 %S (%) | 101.3±35.4 | 77.1±31.4 | <0.001 |

by independent t-test or chi-square test, mean ± S.D. or n (%).

^a^The 16 residential areas of the KNHANES were classified into two groups: urban areas, including metropolitan cities such as Seoul, Busan, Daegu, Incheon, Gwangju, Daejeon, and Ulsan, as well as metropolitan areas such as Gyeonggi province; rural areas, comprising Gangwon, Chungbuk, Chungnam, Jeonnam, Jeonbuk, Gyeongbuk, Gyeongnam, and Jeju provinces. ^b^Household income was assigned to a category according to the following quartiles: 1^st^ quartile (<bottom 25%), 2^nd^ quartile (25-49%), 3^rd^ quartile (50-75%) and 4^th^ quartile (>top 25%). ^c^Occupation group referred to the KSCO-6 classification. Group 1 indicates managers, professionals, technicians and associate professionals; group 2, clerical support workers; group 3, service and sales workers; group 4, skilled agricultural, forestry and fishery workers; group 5, craft and related trades workers, plant and machine operators, and assemblers; group 6, elementary occupations; group 7, housewife, student, and unemployed. ^d^Physical activity of the subjects was categorized according to their participation in recreational physical activity during the week prior to the survey: none, no or minimal activity; mild, >30 minutes of walking more than 5 days per week; moderate, >30 minutes of physical activity in which the subject was tired or breathing slightly hard compared to normal more than 5 days per week; vigorous, >20 min of vigorous physical activity in which the subject was exhausted or breathing hard compared to normal more than 3 days per week. BMI, body mass index; HDL, high density lipoprotein; LDL, low density lipoprotein; BUN, blood urea nitrogen; AST, aspartate aminotransferase; ALT, alanine aminotransferase; FPG, fasting plasma glucose; HOMA2%B, updated homeostasis model assessment for β-cell insulin secretion; HOMA2%S, updated homeostasis model assessment for insulin sensitivity
